# Impact of the Protracted War in Yemen on the Acute Flaccid Paralysis Surveillance System: Retrospective Descriptive Analysis

**DOI:** 10.2196/27638

**Published:** 2021-05-19

**Authors:** Riham Al-Dubaiee, Mutaher AL Qassimi, Ahmed Al-Dar, Abdulwahed Al Serouri, Yousef Khader

**Affiliations:** 1 Ministry of Public Health and Population Sana'a Yemen; 2 Jordan University of Science & Technology Amman Jordan

**Keywords:** acute flaccid paralysis, surveillance indicators, war, Yemen

## Abstract

**Background:**

Highly sensitive acute flaccid paralysis (AFP) surveillance, which includes immediate case investigation and specimen collection, is critical for achieving global polio eradication. In Yemen, the Acute Flaccid Paralysis Surveillance System (AFPSS) was launched in 1998 to achieve the polio eradication target. Although Yemen was certified as a polio-free country in 2009, the protracted war since 2015 has placed the country at risk for polio reemergence.

**Objective:**

The objectives of this analysis were to evaluate the performance of the Yemen AFPSS at both the national and governorate levels, and to assess the impact of the ongoing war on the performance.

**Methods:**

Retrospective descriptive analysis was performed on Yemen secondary AFP surveillance data for the years 2014 (before the war) and 2015-2017 (during the war). Data comprising all children <15 years old reported as having AFP were included in the analysis. AFP surveillance performance was evaluated using World Health Organization–specified AFP surveillance indicators.

**Results:**

At the national level, all indicators were met before and after the war except for “lab results received within ≤28 days,” which was unmet since the war erupted. Furthermore, the indicator “stool specimens arriving at a central level within ≤3 days” was unmet after the war but only in 2017. At the governorate level, although the indicators “adequacy” and “stool specimens arriving at the laboratory in good condition” were met before the war in all governorates, the former indicator was unmet in 9 (41%) governorates since the war erupted and the latter indicator was also unmet in 9 governorates (41%) but only in 2017.

**Conclusions:**

The findings show that some AFP surveillance indicators were negatively impacted by eruption of the war in Yemen due to closure of the Sana’a capital airport and postponement of sample shipment to the reference laboratory, which remained under long-term poor storage conditions. To ensure rapid detection of polio cases, improving specimen collection, storage, and transportation, together with proper and timely shipment of specimens to the reference laboratory should be considered.

## Introduction

### Background

Poliomyelitis is a highly infectious viral disease that affects children below the age of 15 years, and is transmitted from person to person via the feco-oral route [1]. Poliomyelitis is an enteroviral disease that has an incubation period of 9-12 days, and presents as muscle weakness, headache, neck stiffness, fever, nausea, vomiting, and mostly flaccid paralysis [2].

The World Health Assembly adopted a resolution calling for the global eradication of poliomyelitis in 1988. In addition to routine polio immunization included in the Expanded Program of Immunization, two major activities were planned: mass polio vaccination campaigns and surveillance of all cases of acute flaccid paralysis (AFP) [1]. Despite great achievement of polio eradication, transmission has never been stopped in two countries in the eastern Mediterranean region: Pakistan and Afghanistan [3].

In Yemen, AFP surveillance was launched in 1998 to achieve the polio eradication target, and there were no reported cases up to 2004. However, in 2005, 479 cases were reported; therefore, 10 immunization campaigns were launched to halt the outbreak. The last polio case was reported in February 2006, and Yemen was certified as a polio-free country in 2009. Yemen has also experienced three different outbreaks of circulating vaccine-derived polio viruses with 9 cases in April 2011, 4 cases in 2012, 1 case in 2016, and, most recently, one case reported in September 2020 [4-6]. Although the country is currently free of poliovirus, it is considered one of the high-risk countries for reemergence of the virus due to the current war situation.

### The Yemen Acute Flaccid Paralysis Surveillance System

The Yemen Acute Flaccid Paralysis Surveillance System (AFPSS) was launched in 1998 with strong support from the World Health Organization (WHO) to help in the early detection of any AFP case and to ensure immediate notification. The AFP surveillance data are regularly shared with the WHO country and regional offices, and with the Global Polio Eradication Initiative. As Yemen does not have a national polio laboratory, Kenya Medical Research Institute in Nairobi has served as a reference laboratory for Yemen since 2015. The AFP surveillance indicators were adapted from WHO certification standards [7].

Nationally, the AFPSS consists of a national AFP coordinator and four assistants. At the level of governorates, there are 35 coordinators and their assistants. In all districts, there are approximately 333 coordinators and 1980 officers in health facilities.

The purposes of the system include early detection of any AFP case and investigating it, evaluation of the polio eradication program performance, and certifying and confirming that Yemen is still free of poliovirus.

Since 2015, the war has affected the performance of the health system in Yemen, with nearly half of all health facilities suffering damage or unable to function because of severe shortages of staff and equipment. Consequently, an estimated 56% of the population does not have regular access to basic health care [8].

### Aim

The objectives of this analysis were to evaluate the performance of the Yemen AFPSS at both the national and governorate levels, and to assess the impact of the ongoing Yemeni war on the performance.

## Methods

A retrospective descriptive analysis was performed on secondary AFP surveillance data for Yemen for the years 2014 (before the war) and 2015-2017 (during the war). The data included all children <15 years old who were reported in the AFSSP as an AFP case. AFP surveillance performance was evaluated using the following WHO-specified AFP surveillance indicators [7]: (1) nonpolio AFP rate in children ≤15 years of age (target: ≥2/100,000) annually and investigation within less than 48 hours of the report (target: ≥80%); (2) two stool specimens collected at least 24 hours apart and within 14 days of paralysis onset (target: ≥80%), reflecting the adequacy rate; (3) stool specimens arriving at the central level within ≤3 days (target: ≥80%); (4) stool specimens arriving at the laboratory in “good condition” (target: ≥80%); (5) notification within ≤7 days of paralysis onset (target: ≥80%); (6) nonpolio enterovirus isolation rate (target: ≥10%) to reflect the lab’s performance level and virus detection sensitivity; and (7) lab results received within ≤28 days (target: ≥80%).

The study was performed from October to January 2019, including data from 2014 to 2017. The data were entered and analyzed in Microsoft Excel.

## Results

### AFP Surveillance Performance Indicators at the National Level

Table 1 shows the AFP surveillance performance indicators at the national level for 2014-2017. All indicators met the targets in 2014 before the war. After the war erupted, all indicators were met in 2015 and 2016 except for the “lab results received within ≤28 days.” Furthermore, in 2017, the indicators “lab results received within ≤28 days” and “stool specimens arriving at the central level within ≤3 days” were not met.

**Table 1:** Acute flaccid paralysis (AFP) surveillance performance indicators at the national level, 2014-2017.

Indicator	Target	Reported value			
		2014	2015	2016	2017
Nonpolio AFP rate in children ≤15 years old	≥2/100,000	4.6	4.2	5.2	5.2
Investigation within ≤48 hours of the report	≥80%	98%	99%	100%	100%
Two stool specimens collected at least 24 hours apart within 14 days of paralysis (adequacy)	≥80%	95%	91%	91%	82%
Stool specimens arriving at a central level within ≤3 days	≥80%	88%	85%	83%	78%^a^
Stool specimens arriving at the reference laboratory in good condition	≥80%	99%	99%	99%	81%
Notification within ≤7 days of paralysis onset	≥80%	86%	85%	85%	87%
Nonpolio enterovirus isolation rate	≥10%	18%	19%	22%	15%
Lab results received within ≤28 days	≥80%	83%	48%^a^	24%^a^	33%^a^

^a^Did not meet target.

### AFP Surveillance Performance Indicators at the Governorate Level

The targets for the two indicators “nonpolio AFP rate in children ≤15 years” and “investigation within ≤48 hours of the report” were met in all governorates in all years both before and after the war. Although the adequacy indicator “two stool specimens collected at least 24 hours apart within 14 days of paralysis” met the target in all governorates in 2014 (before the war), the target was not met in two governorates in 2015 (Abyan and AlDhale’e) and 2016 (AlDhale’e and Socotra), and in seven governorates (AlBayda, AlJawf, Amran, Ibb, Raymah, Sana’a city, and Socotra) in 2017.

The target for the indicator “stool specimens arriving at the central level within ≤3 days” was not met in 5 governorates in 2014, 7 governorates in 2015, 11 governorates in 2016, and 14 governorates in 2017. The indicator “stool specimens arriving at the reference laboratory in good condition” met the target in all governorates in all years except for 2017, in which 9 governorates (AlBayda, AlJawf, Almahra, Amran, Dhamar, Lahj, Raymah, Sana’a city, and Taizz) did not meet the target for this indicator.

A total of 5 governorates in 2014 (AlHudaydah, Almahweet, Amran, Hajjah, and Sa’ada) and 2015 (Abyan, AlHudaydah, AlJawf, Hajjah, and Sa’ada), 6 governorates (Almahweet, Amran, Ibb, Sa’ada, Socotra, and Taizz) in 2016, and 7 governorates (AlBayda, AlHudaydah, AlJawf, Amran, Hajjah, Sa’ada, and Socotra) in 2017 did not meet the target of the indicator “notification within ≤7 days of paralysis onset.” The targets for the indicator “nonpolio enterovirus isolation rate” had not been met in 3 governorates (Abyan, Shabwah, and Taizz) in 2014, in 4 governorates (Aden, AlHudaydah, Almahra, and Sa’ada) in 2015, 5 governorates (Abyan, Almahra, Almahweet, Hadramaut Sayoun, and Shabwah) in 2016, and 4 governorates (AlBayda, Sa’ada, Sana’a city, and Socotra) in 2017. The target for the indicator “lab results received within ≤28 days” did not meet the target in 7 governorates only in 2014 (before the war) and in all governorates in all years after the war.

## Discussion

### Principal Findings

Evaluation of a surveillance system is important to examine the operation of the system and to show how it adheres to implementation protocols. Thus, the current AFPSS evaluation will help to identify whether the system met its target indicators properly.

This evaluation showed that at the national level, all indicators were met before the war erupted in 2014; however, the indicator “lab results received within ≤28 days” was not met through 2015-2017 after the war. This is because of closure of the main national airport in Sana’a in 2015, which remains closed to date [9]. Therefore, sending the samples to the reference laboratory in Kenya was not possible and the lab results were not received in a timely manner.

The indicator “stool specimens arriving at the central level within ≤3 days” was not met at the national level during 2017, which was most likely due to the lack of funding available for transporting specimens from the governorates to the national level, as well as the disruption and danger of some roads [8]. A similar result was reported from Iraq after the war erupted [10]. At the governorates level, we found that the number of governorates that did not meet this target increased as the war continued, which is attributed to the same reasons mentioned above.

The target for the indicator “nonpolio AFP rate in children ≤15 years old” was met at the national level and for all governorates in all years, and even exceeded the WHO-established minimum nonpolio AFP rate. Thus, the sensitivity of AFP surveillance has been good before and after the war. A similar finding was reported from Nigeria, the eastern Mediterranean region, and other WHO regions [10-13]. The lack of an effect of war on this indicator has been found for other countries in conflict, such as Iraq [10]. Similarly, the indicator “investigation within 48 hours of report of cases” met the target at the national level and in all governorates in all years, and was not affected by war in this study, in line with findings from other countries in conflict, such as Iraq [10].

The adequacy indicator also met the target in all years at the national level before and after the war erupted. The target of this indicator was met in similar evaluations in other countries, including Iraq and Nigeria [10-12]. This reflects a better awareness by patients’ families and medical caregivers regarding the importance of early AFP case detection. Although this indicator was met at all governorates before the war, it was not met in some governorates after eruption of the war, which may be due to a discontinuation of training. Lack of training was also found to be the reason for not meeting the adequacy target in other countries such as Zimbabwe [14].

The indicator “stool specimen arriving at the reference laboratory in good condition” met the target at the national level in all years. This indicator also met the target in all governorates and all years of evaluation except for 2017. This was most likely due to suspension of the shipment of specimens to the reference laboratory for 3 months that were left under a poor storage condition in 2017. Furthermore, the indicator “notification within 7 days of paralysis onset” was met at the national level in all years. However, the target was not met in some governorates because of poor knowledge about the timeliness of notification and the lack of training at this time.

Although the indicator “nonpolio enterovirus isolation rate” met the target at the national level in all years, it was not met in some governorates before as well as after eruption of the war. This may reflect the poor collection and transport of specimens due to reduced training as well as accessibility and transport problems.

### Conclusions

The findings of this study showed that some AFP indicators were negatively impacted by eruption of the war in Yemen due to closure of the Sana’a capital airport and postponement of specimen shipment to the reference laboratory, leaving specimens in a poor storage condition. Therefore, proper and timely shipment of specimens to the reference laboratory in Kenya as well as ensuring receiving the lab results within 28 days is strongly recommended. It is also important to ensure the availability of funds for the transport of specimens from governorates to the national level, and to continuously train health workers on the proper collection and transport of specimens to ensure achievement of AFP indicators.

## Abbreviations

AFP: acute flaccid paralysis
AFPSS: Acute Flaccid Paralysis Surveillance System
WHO: World Health Organization
